# What Is in a Simplicial Complex? A Metaplex-Based Approach to Its Structure and Dynamics

**DOI:** 10.3390/e25121599

**Published:** 2023-11-29

**Authors:** Manuel Miranda, Gissell Estrada-Rodriguez, Ernesto Estrada

**Affiliations:** 1 Institute of Cross-Disciplinary Physics and Complex Systems, IFISC (UIB-CSIC), 07122 Palma de Mallorca, Spain; mmiranda@ifisc.uib-csic.es; 2 Departament de Matemàtica, Universitat Politècnica de Catalunya, 08034 Barcelona, Spain; gissell.estrada@upc.edu

**Keywords:** simplicial complex, geometric realization, metaplexes, diffusion, brain networks, higher-order networks

## Abstract

Geometric realization of simplicial complexes makes them a unique representation of complex systems. The existence of local continuous spaces at the simplices level with global discrete connectivity between simplices makes the analysis of dynamical systems on simplicial complexes a challenging problem. In this work, we provide some examples of complex systems in which this representation would be a more appropriate model of real-world phenomena. Here, we generalize the concept of metaplexes to embrace that of geometric simplicial complexes, which also includes the definition of dynamical systems on them. A metaplex is formed by regions of a continuous space of any dimension interconnected by sinks and sources that works controlled by discrete (graph) operators. The definition of simplicial metaplexes given here allows the description of the diffusion dynamics of this system in a way that solves the existing problems with previous models. We make a detailed analysis of the generalities and possible extensions of this model beyond simplicial complexes, e.g., from polytopal and cell complexes to manifold complexes, and apply it to a real-world simplicial complex representing the visual cortex of a macaque.

## 1. Introduction

The complexities of many systems, generically known as complex systems, are manifested not only in their structures and behavior [1,2], but also in the difficulties of defining precisely what they are (see [3] and references therein) and, moreover, by the several ways of representing them [4]. Due to their networked nature, the use of graphs has been ubiquitous in representing complex systems [5,6]. However, other representations, including temporal networks [7], multiplexes and multilayers [8,9], hypergraphs [10,11], and simplicial complexes [12,13,14], have been claimed as (more) appropriate than simple graphs for (certain) classes of systems [15]. Certainly, the use of these representations constitutes an advancement in our understanding of the structure and dynamics of complex systems. But, unfortunately, their abuse may also represent a drawback to the necessary understanding of complex systems in the real world.

Currently, we are living on the crest of a wave on the use of hypergraphs and simplicial complexes to study man-made and natural complex systems. Nowadays, the term “higher-order networks” has been coined to group these representations [16,17,18,19,20,21,22]. To motivate the necessity of using these higher-order structures let us consider the relations of coauthorship of scientific papers. These systems have been extensively studied as networks, representing authors and coauthorship as the nodes and edges of the graph, respectively [23,24]. However, sometimes coauthorship goes beyond the pairwise relations represented in a graph, and *k*-cliques (of different sizes) of coauthors may coexist in the same system [25]. This situation can be represented by a hypergraph in which pairs, triples, etc., are grouped together in hyperedges [10]. It is frequent in coauthorship networks that one author is a single author of a paper, as well as a coauthor of other papers with one, two, or more coauthors [25]. This is also illustrated by a hypergraph in which a single node would participate in hyperedges for different cardinalities.

Some confusion may have emerged in the field of higher-order networks due to the fact that a hypergraph like the one described before, where the set of hyperedges is closed under inclusion, is also known as an abstract simplicial complex [16]; that is, in an abstract simplicial complex, every nonempty subset of a hyperedge is also a hyperedge. Therefore, some of the claims on the use of simplicial complexes for representing complex systems reduce to the particular case of specific hypergraphs, i.e., purely combinatorial objects, where the set of hyperedges is closed under inclusion. It seems that the use of simplicial complexes predates that of hypergraphs for studying complex systems. It was the British mathematician Ronald H. Atkin in as early as 1972 who proposed the abstract simplicial complex “as the vehicle for that sense of structure which is inherent in either the laws of physics or the behavior of social systems” [26] (see also [27]). Almost a decade later, another mathematician Stephen B. Seidman proposed the use of hypergraphs to “permit the study of structure induced by non-dyadic relationships” [28]. As resumed by Freeman and White [29], the work of Seidman showed that the hypergraphs “could be used to provide a similar–and perhaps simpler–representation of two mode data” than the one of Atkin. Nowadays, hypergraphs are a powerful tool to represent and analyze data (see, for instance, [30,31]).

However, one important step forward was already made in Atkin’s 1972 paper when the author stated that “it is often helpful to think of a geometric realization of the complex” such that every simplex is a closed convex polyhedron in a suitable space [26]. In this sense, informally (see further for a formal definition), a simplicial complex is a space with a triangulation, that is, a topological space made of vertices, edges, triangles, tetrahedra, and higher-dimensional equivalents connected to each other by their edges, vertices, faces, and so on. The main distinctive characteristic of a geometric simplex relative to other discrete objects is that the first encloses a continuous space. For instance, we can represent the connection between two airports by an edge, which indicates that there are flights that depart from one of the airports and arrive at the other. We cannot trace the position of a flight between the two airports, such information simply does not exist in this representation. However, if we represent the trajectory of a flight in space, we are connecting the two vertices by a 1-simplex, which describes the continuous space traveled by a plane between the two airports. In the geometric simplex, we also kept that the relations are closed under inclusion. For instance, in a 2-simplex (triangle) every of the three edges (1-simplices) are also part of the simplex, as well as the vertices (0-simplices).

Although the geometric simplicial complex could be very appealing, as claimed by Atkin [26,27] and more recently by others [21,22,25,32,33,34], a problem may arise when considering dynamics on them. If a dynamics simulating the spreading of information using an epidemiological model is defined on top of the geometric simplicial complex, we will find probabilities of getting infected for any region of the continuous space; that is, in a 2-simplex, not only the three individuals can be infected or receive information, but such information or probability of infection exists also for any point in the whole triangle delimited by the three individuals. This is hard to digest without invoking mystical ideas, and the problem is not limited to the propagation of information but to most dynamical systems that we can define on a geometric simplicial complex.

There are, however, systems in which the use of geometric simplicial complexes could be necessary for understanding some of the dynamics occurring on them. These are the cases, for instance, of (i) protein residue networks, where the interaction between amino acids generates (hydrophobic and electrostatic) fields filling the inter-residue space; (ii) protein–protein interactions, where the proteins form complexes of individual proteins glued together by noncovalent interactions; (iii) landscape networks, where very close patches can allow the diffusion of species across the areas delimited by them; networks of fractures in rocks, where close enough fractures may allow the diffusion of material through a porous continuous space; neuronal networks, where volume transmission (see further) can spill over neurotransmitters to the extracellular space between neurons, among others.

Here, we will focus on geometric simplicial complexes and will use the term simplicial complex for short. In the case of combinatorial relations, such as the ones between coauthors, which are closed under inclusion, we do not see any reason to call them a simplicial complex but a hypergraph as there is nothing geometric in such representation.

## 2. On the Problem of Representation

We illustrate the representation problem of a complex system by means of the example of a neuronal system. For the sake of simplicity, we consider three neurons having synapses among them. There are two different types of transmission mechanisms through a pair of neurons. The first is the most common one and it is known as wiring transmission (WT) [35]. This refers to the mode of intercellular communication in which the existence of a virtual wire connecting the cell source of the signal (message) with the cell target of the signal exists. It is typical of the electrical synapses but also of the chemical ones. However, in the last case, a second type of inter-neuron transmission may occur: volume transmission (VT) [35,36,37,38,39]. It refers to the mode of intercellular communication that occurs through the extracellular fluid (ECF) and in the cerebrospinal fluid (CSF) of the brain; that is, during chemical synapses, amounts of neurotransmitters are spilled over to the ECF near the perisynaptic region, such that VT signals move from source to target cells via energy gradients leading to diffusion and convection.

**Figure 1:**
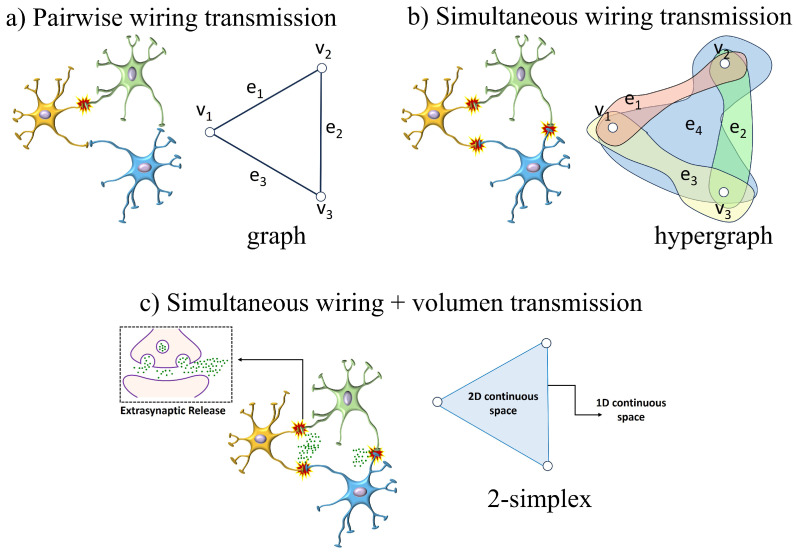
Schematic illustration of three different representations of the interaction between three neurons. (**a**) A graph representation of the system when wiring transmission (WT) alone is considered between pairs of neurons. (**b**) A hypergraph representation of the system when simultaneous WT may occur not only in a pairwise way but among *k*-ary groups. (**c**) In the case of chemical synapses, WT may coexist with volume transmission (VT) produced by the spillover of neurotransmitters to the extracellular space. In this case, the representation of the system as a simplex is more appropriate (see text for details).

If we are interested in analyzing the WT between pairs of neurons in the system, we are in the presence of a scenario like the one illustrated in Figure 1a. In this scenario, it is enough to use a representation of the system as a (weighted, directed) graph. A (weighted) graph $G=\left(V,E,\phi ,W\right)$ is formed by a set of vertices $V=\left\{v_{0},\ldots ,v_{k}\right\}$ and a set of edges $E=\left\{\left.\left(v_{i},v_{j}\right)\right|v_{i},v_{j}\in V\right\}$ [6]. Then, a set of edge weights *W* may represent a characteristic feature of the synaptic connection, which are mapped onto the edges by the mapping: ϕ:W→E. Note that the vertex index starts from 0 instead of 1. This choice is to make the vertex of the graph to follow the same index as the vertex of the simplicial complexes, which will be defined later.

It is plausible that the synapses between neurons occur in a simultaneous way including not only pairwise interactions between neurons but also by triples, quadruples, etc., as illustrated in Figure 1b. In this scenario, the previous graph-theoretic representation is no longer appropriate. To capture these new types of interactions we need an extension of the graph known as hypergraph [40]. In the simple hypergraph H=(V,E), *V* continues to be the set of nodes, but now, *E* is the set of hyperedges. A hyperedge is any subset of nodes in *V*. Thus, the set of hyperedges can be seen as the subset of the power set of *V*, $E\subset P\left(V\right)$.

We remark that the hypergraph, like the graph, is a discrete system; that is, although we have drawn the hyperedges as colored contours that include the set of vertices in the hyperedge, they do not mean any kind of “physical” space. These hyperedges indicate only that a relation (binary in graphs or *k*-ary in hypergraphs) exists among the group of vertices. Therefore, in diffusive dynamics taking place on the hypergraph, we cannot find the diffusive particles on the (hyper)edges of the (hyper)graph. The dynamics occur as if the particles are annihilated at a given vertex and created at the others.

So far we have mentioned only the WT among the neurons of the system, but when VT is considered the situation changes dramatically. Let us consider that some amount of the chemicals transmitted between two neurons is spilled over through the ECF in the perisynaptic region and that such neurotransmitter is recaptured by another neuron. We are no longer in the presence of a discrete relation between the three neurons. The intercellular region, bounded by the three neurons, forms a continuous space, which can be approximated by the triangle between the three neurons. VT is always accompanied by WT. This means that not all the neurotransmitters are spilled over to the perisynaptic region, but some of them are diffused through the wiring connection between the two neurons. Therefore, we should be able to trace back the concentration of the neurotransmitter through the path connecting the two neurons. In other words, we have a one-dimensional space connecting the pairs of neurons and a two-dimensional continuous space between the triple of neurons. Similarly, we can extend this idea to consider three-dimensional regions of continuous space. This new scenario cannot be appropriately described by the graph or by the hypergraph. A new type of representation, like the one illustrated in Figure 1c is needed. It is known as a simplex [41].

Formally, a simplex is defined as follows:

**Definition** **1.** *A set of points $s=\left\{a_{0},a_{1},\ldots ,a_{k}\right\}$ in $R^{n}$ (k≥n≥2) is geometrically independent if the set $\left\{a_{1}-a_{0},a_{2}-a_{0},\ldots ,a_{k}-a_{0},\right\}$ is linearly independent in $R^{n}$. By definition, any singleton is geometrically independent.*

**Lemma** **1.** *Let $s=\left\{a_{0},a_{1},\ldots ,a_{k}\right\}\subseteq R^{n}$ be geometrically independent. Then, there is a unique k-dimensional hyperplane in $R^{n}$ that contains s.*

**Remark** **1.** *Any set $s=\left\{a_{0},a_{1}\right\}$ is geometrically independent in $R^{n}$. A set $s=\left\{a_{0},a_{1},a_{2}\right\}\subset R^{n}$, for n≥2, is geometrically independent if and only if they are the vertices of a triangle. A set $s=\left\{a_{0},a_{1},a_{2},a_{3}\right\}\subset R^{n}$, for n≥3, is geometrically independent if and only if these points are the vertices of a tetrahedron. Generally, a set $s=\left\{a_{0},\cdots ,a_{m}\right\}\subset R^{n}$, for n≥m, is geometrically independent if these points are the vertices of a polytope.*

**Definition** **2.** *Let $s=\left\{a_{0},a_{1},\ldots ,a_{k}\right\}\subseteq R^{n}$ be geometrically independent. Then, the set*

(1) $$\begin{array}{cc} s_{k} & =\left(a_{0},a_{1},\ldots ,a_{k}\right)\\ \; & =\left\{\sum_{i=0}^{k}\lambda_{i}a_{i}:\sum_{i=0}^{k}\lambda_{i}=1,\lambda_{i}\in R,\lambda_{i}>0,i=0,\ldots ,k\right\}, \end{array}$$

*is called a k-simplex with vertices given by $a_{i}$.*

**Lemma** **2.** *Let $s=\left\{a_{0},a_{1},\ldots ,a_{k}\right\}\subseteq R^{n}$ be geometrically independent. Then, the simplex $s_{k}$ is a convex subset of $R^{n}$.*

A face τ of a simplex $s_{k}$ is a linear space spanned by proper subsets of vertices of $s_{k}$.

Of course, we can extend the scheme of the three neurons to an entire web of inter-neuron interactions. In this case, we will have a set of simplices which are mutually connected. This system is then known as a simplicial complex, and it is formally defined below.

**Definition** **3.** *Let K be a collection of finitely many simplices. Then, K is a simplicial complex if the following conditions are satisfied:*

*1. If $s_{k}\in K$ and τ is a face of $s_{k}$, then this implies that τ∈K.*

*2. If $s_{k},\tau \in K$, and $s_{k}\cap \tau \neq Ø$, then this implies that $s_{k}\cap \tau$ is a common face of $s_{k}$ and τ.*

These properties are important in the field of algebraic topology, as they allow the definition of a homology and use powerful tools such as the Hodge decomposition theorem [42]. Hence, the concept of abstract simplicial complex [43] (p. 153) was created to denote any family of sets with such property, without requiring any geometrical property.

In the study of complex systems, it is frequent to use the term simplicial complex to denote hypergraphs whose hyperedges are closed by inclusion [44]. In such cases, these objects refer to abstract simplicial complexes where geometric properties are not explicitly considered.

## 3. What Is a Metaplex?

In the representations of complex systems as graphs and hypergraphs, the emphasis is placed on the patterns of connectivity between the entities of the system. In these representations, the nodes are reduced to a simple, structureless, point. However, the internal structure of complex entities, e.g., neurons, individuals, landscape patches, etc., plays a fundamental role in the evolution of the dynamics of these systems. With this in mind, some of the current authors have developed the concept of “metaplex” [45]. From a graph-theoretic point of view, a metaplex can be seen as a graph in which each vertex has a continuous structure, instead of being a structureless point. Additionally, a metaplex can be seen from the perspective of Differential Geometry as a collection of domains in which there are different regions that are coupled to other domains in the collection. We now proceed to define this concept in a rigorous way.

**Definition** **4.** *A metaplex is a 5-tuple Y=(V,E,ω,I,F), where (V,E) is a graph, $\omega =\{\Omega_{i}\;|\;i=0,\cdots ,k\}$ is a collection of domains, $\Omega_{i}\subset R^{n_{i}}$, where $n_{i}\in N$ for each i, I:V→ω and $F=\{F_{ij}\;|\;i,j=0,\cdots ,k;\;i\neq j\}$ are bounded analytic maps between these domains. This is, for 0≤i≠j≤k, $F_{ij}:\Omega_{i}\to \Omega_{j}$, $∥F_{ij}{∥}_{\infty }<\infty$.*

Here, the underlying graph of the metaplex is the set $\left(V=\left[0,\ldots ,k\right],E=\{\left[i,j\right]:F_{ij}\neq Ø\}\right)$. Then, the tuple (ω,I) consists of the set of domains $\left(\Omega_{i}\right)$ without repetitions, and I assigns to each node i∈[0,…,k] the element on the set ω corresponding to the original $\Omega_{i}$. We have modified here the previous definition of metaplex [45] to remark on an important topological characteristic of this mathematical object: sinks and sources.

**Definition** **5.** *Let Y=(V,E,ω,I,F) be a metaplex. Then, for each 0≤i,j≤k we call $\mathrm{dom}\left(F_{ij}\right)\subseteq \Omega_{i}$ a sink of the domain $\Omega_{i}$ connected to Im(Fij)⊆Ωj, which is a source of the domain $\Omega_{j}$.*

These pairwise connected regions relate the different domains, coupling the two (continuous) spaces in a similar way as edges relate the discrete nodes in a graph.

## 4. Diffusion on Graphs, Simplicial Complexes and Metaplexes

### 4.1. Diffusion on Graphs

Diffusive processes are ubiquitous in man-made and natural complex systems. Due to the networked nature of these systems, it is frequent to analyze the diffusion dynamics on a graph [46]. For the sake of completion, we state here the main aspects of the diffusion dynamics on simple graphs. Let L=K−A be the Laplacian matrix of the graph, where *K* is a diagonal matrix of vertex degrees and *A* is the adjacency matrix of the graph. Notice that if ∇ is the vertex-edge oriented incidence matrix (gradient) of the graph, then $L=\nabla \nabla^{T}$. Then, the change in the concentration of an item at the nodes of the graph is accounted for by the vector $\dot{x}\left(t\right)$ and described by the diffusion equation on graphs:

(2) $$\dot{x}\left(t\right)=-\gamma Lx\left(t\right),\;x\left(0\right)=x_{0},$$

where γ is the diffusivity coefficient, hereafter taken as unity. The solution of this equation is given by the heat kernel $e^{-tL},$ such that $x\left(t\right)=e^{-tL}x_{0}$. Let $\mu_{0}\leq \mu_{1}\leq \cdots \leq \mu_{k}$ be the eigenvalues of *L* and $\psi_{j}$ the column eigenvector of *L* corresponding to $\mu_{j}$. Then, the solution of the diffusion equation on the graph can be written as:

(3) $$x\left(t\right)=e^{-t\mu_{0}}\left(\psi_{0}^{T}x_{0}\right)\psi_{0}+e^{-t\mu_{1}}\left(\psi_{1}^{T}x_{0}\right)\psi_{1}+\cdots +e^{-t\mu_{k}}\left(\psi_{k}^{T}x_{0}\right)\psi_{k}.$$

It is easy to see that $\mu_{0}=0$ and that its multiplicity are equal to the number of connected components of the graph. Therefore, in a connected graph, when t→∞:

(4) $$x\left(t\right)\to \left(\psi_{0}^{T}x_{0}\right)\psi_{0}=\frac{1}{k}\sum_{i=0}^{k}x_{0}\left(i\right).$$

**Figure 2:**
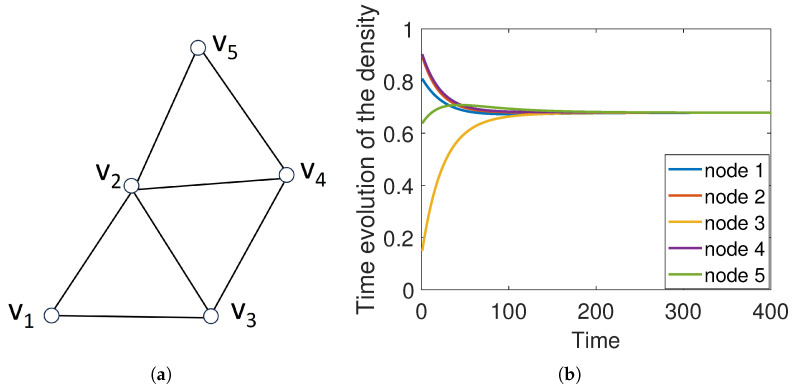
Illustration of a simple graph (**a**) and a diffusion process taken place on it with a random initial condition (**b**).

This characteristic feature of diffusion is very important for the further analysis of these dynamics on simplicial complexes; that is, standard diffusion is a conservative consensus process in which the entities of the system end up at an equilibrium state, which is the average of their initial states. The rate of convergence depends on the algebraic connectivity of the graph: $\mu_{1}$. An example is provided in Figure 2.

### 4.2. Diffusion on Simplicial Complexes

A way to consider diffusive dynamics on a simplicial complex is to represent it by means of its incidence matrices $B_{m}$ for 1≤m≤M. Here, *M* is the dimension of the simplicial complex, namely the highest dimension among the simplices composing the complex. The matrix $B_{1}$ is the usual incidence matrix of a graph, with dimension equal to the number of nodes times the number of edges, which is constructed as follows.

Let i<j be two nodes of the simplicial complex so that the edge [i,j] is also in the simplicial complex. Then, ${\left(B_{1}\right)}_{i,[i,j]}=-1$, ${\left(B_{1}\right)}_{j,[i,j]}=1$ and ${\left(B_{1}\right)}_{l,[i,j]}=0$ for *l* any other node in the simplicial complex. Note that the choice of the sign depends on the labels assigned to the nodes, so the same graph may be represented by different incidence matrices.

In general, the incidence matrix of order *m*, $B_{m}$, has a dimension equal to the number of (m−1)-simplices in the simplicial complex multiplied by the number of *m*-simplices. The entry that corresponds to the (m−1)-simplex $\left[i_{1},\ldots ,i_{m-1}\right]$ and the *m*-simplex $\left[i_{1},\ldots ,i_{m-1},j\right]$ is equal to the sign of the permutation that orders the set $\left\{i_{1},\ldots ,i_{m-1},j\right\}$ into an increasing set.

Then, we can define the *m*-th order Laplacian as $L_{m}=B_{m}^{⊤}B_{m}+B_{m+1}B_{m+1}^{⊤}$ for 0≤m≤M, assuming the notation $B_{0}=Ø=B_{M+1}$. The case $L_{0}=B_{1}^{⊤}B_{1}$ is the usual graph Laplacian. These *m*-th order Laplacians act over the *m*-dimensional simplices in the complex that are connected via m−1 or m+1-dimensional simplices. Thus, we can define the total Laplacian L as the block diagonal matrix consisting of the *m*-th Laplacians, $L={\left(L_{m}\right)}_{m=0}^{K}$, which acts over vectors that take values on each of the simplices forming the complex.

**Figure 3:**
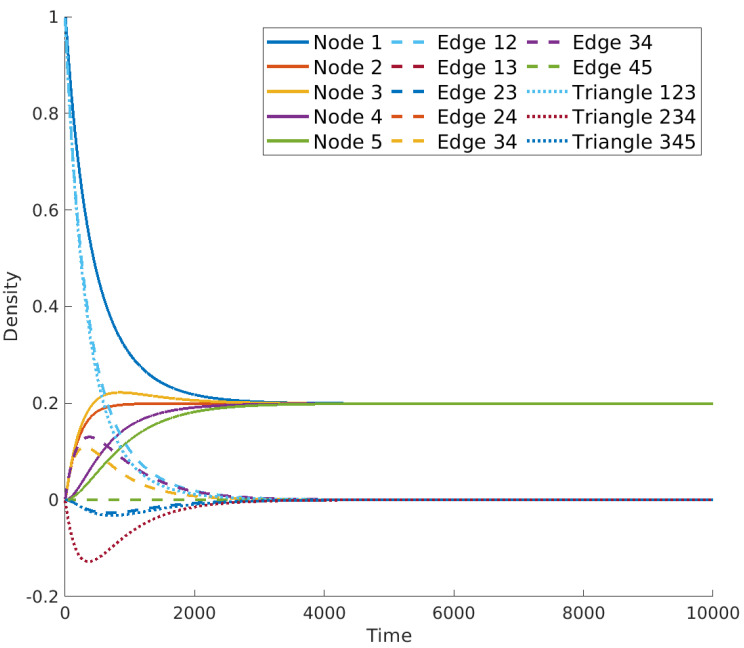
Evolution of the diffusion equation on the simplicial complex Figure 4 using operator L.

These operators were studied in [47], where the authors remarked on some relevant properties, such as the positive semidefiniteness, but also showed several problems. The first one is that the number of repetitions of the zero eigenvalues of the *m*-th Laplacian equals the Betti number $\beta_{m}$. For the usual Laplacian $L_{0}$, the associated Betti number $\beta_{0}$ equals the number of connected components in the graph, thus assuring the existence of a non-trivial steady state for the equation $\dot{x}\left(t\right)=-L_{0}x\left(t\right)$. On the other hand, there are simplicial complexes for which $\beta_{m}=0$ for some *m*, which means that the equation $\dot{x}\left(t\right)=-L_{m}x\left(t\right)$ only accepts the trivial solution x=0 for large times whatever is the initial condition (see Figure 3). This problem was patched in the same article [47] by proposing the use of $L-\lambda_{1}v_{1}v_{1}^{T}$ as diffusion operator, where $\lambda_{1}$ is the first non-zero eigenvalue and $v_{1}$ its associated eigenvector.

**Figure 4:**
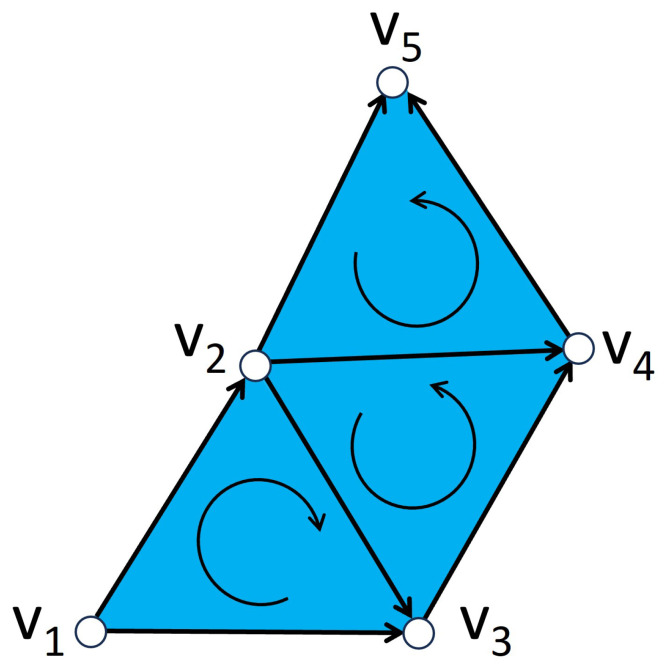
Example of the simplicial complex used by [47].

Nevertheless, modeling diffusion in this way gives rise to other problems. For instance, even for positive initial conditions, the system may evolve by taking negative values; that is, there are negative concentrations of the entities of the system at certain times, which should not happen in a model of a diffusive process. For instance, in the simplex shown in Figure 4, we ran the equation $\dot{x}\left(t\right)=-Lx\left(t\right)$ with initial condition $u\left(0\right)=u_{0}=1$ in the node $V_{1}$, in the edge (1,2) and in the triangle (1,2,3), and 0 elsewhere. Figure 3 shows the results, where it can be observed that the concentration in the edge (2,3) and in the triangle (3,4,5) take negative values at the beginning of the simulation. Also worrying is the fact that there is not a global steady state for diffusion in the simplicial complex, but the nodes reach an independent consensus state from the steady states reached by edges and triangles, which is equal to 0 in this case. This should not happen in connected simplicial complexes like the one studied here. Again, we emphasize that in the standard diffusion process, the steady state is a global consensus of the system given by the average of the initial condition. If we were modeling the diffusion of temperature in the previous example, the outcome would be that the system arrives at a state in which one part has a temperature and another part has a different one, never reaching a common temperature.

Moreover, the higher-order Laplacians only reflect changes between simplices of the same order, so the values of the concentration over the simplices of higher and/or lower order connected to them do not affect the value of the concentration of the original simplex. As an example, consider two 0 simplices (nodes) *i* and *j* connected by the 1-simplex (link) l=[i,j]. Let $x=(x_{i},x_{j},x_{l})$ be a vector of concentrations on this simplicial complex satisfying equation $\dot{x}\left(t\right)=-Lx\left(t\right)$. Then, changes in the values of $x_{i}$ produce changes in the values of $x_{j}$ thanks to the dynamical equation, but will not affect $x_{l}$ in any way, although the node *i* is connected to the link *l* and the changes in node *j* happen through the link *l*.

### 4.3. Diffusion on Metaplexes

To describe a diffusion on a metaplex, let u(x,t) be the density of the diffusive particle at time *t* for x in a domain in $R^{n}$. Then, a dynamical system on a metaplex [45] is defined as follows.

**Definition** **6.** *A dynamical system on a metaplex is a tuple (H={Hi:L2(Ωi)→L2(Ωi)},T={Tij:L2(dom(Fij))→L2(Im(Fij))}) such that, for any $u_{0}={\left({\left(u_{0}\right)}_{i}\right)}_{i=0}^{k}\in {\left(L^{2}\left(\Omega_{i}\right)\right)}_{i=0}^{k}$, the initial value problem $\partial_{t}u_{i}\left(t\right)=H_{i}\left(u_{i}\left(t\right)\right),u_{i}\left(0\right)={\left(u_{0}\right)}_{i}$ is well-posed.*

Each of the elements in the tuple is related to one of the continuous or discrete points of view that metaplexes join together. Each of the operators in H is a continuous differential operator describing the process on the space $\Omega_{i}$, such as the Laplacian. On the other hand, T can be seen as the matrix representing a discrete operator describing the process among the nodes $\Omega_{i}$, such as the graph Laplacian; thus, it is compact and linear.

We can see the previous definition as a system of coupled differential equations for the density $u_{i}\left(x,t\right)$ in the node $v_{i}\in V$, as in [45], provided that we extend all functions $F_{ij}$ as zero outside of the sink–source,

(5) $$\partial_{t}u_{i}\left(x,t\right)=H_{i}\left(u_{i}\left(x,t\right)\right)-\sum_{j=0}^{k}T_{ij}\left(u_{i}\left(x,t\right)\right)+\sum_{j=0}^{k}T_{ji}\left(u_{j}\left(F_{ji}^{-1}\left(x\right),t\right)\right),\;x\in \Omega_{i}.$$

The conditions on the sets of operators H and T allow us to know that analytic solutions should exist by the Cauchy–Kovalevski theorem [48].

These definitions allow extending both the concept of dynamical systems to graphs and to continuous domains. A dynamical system on a graph is equivalent to a dynamical system on a metaplex where all the domains $\Omega_{i}$ are points, $F_{ij}$ are the weights of the edges, and the dynamical system consists of $H_{i}$ being the identity on the points with $T_{ij}$ being the entries of the matrix operator on the graph. The case of a dynamical system on a continuous domain is even simpler, as it would consist only of one domain $\Omega_{1}=\Omega$ with operator $H_{1}=H$ and, as there are no other domains to which it is connected, there are neither $F_{ij}$ nor $T_{ij}$.

**Figure 5:**
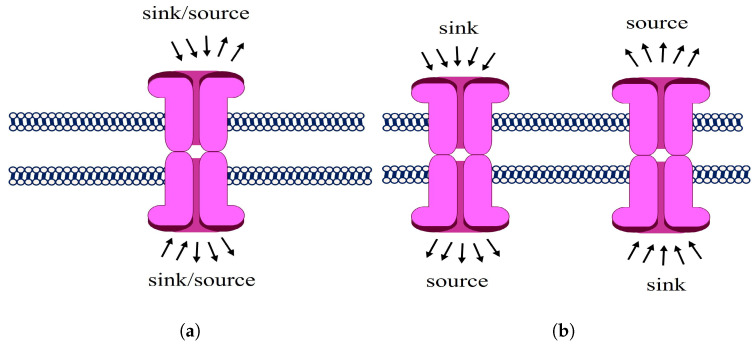
Example of two metaplexes whose transition matrices are identical but do not show the same dynamical behavior due to the position of the sinks and the sources. (**a**) The sink and source coincide in the same place. (**b**) The sink and source are spatially separated inside the continuous space. If we assume that both sink–source connections are equal, the transition matrix is given by $T=\left(\begin{array}{cc} -1 & 1\\ 1 & -1 \end{array}\right)=-L$.

Moreover, it is important to note some differences that arise when combining these two points of view. First, it is the change between T and a transition matrix in a graph due to the sink–source relation. Similarly to the adjacency matrix of the underlying graph of the metaplex, we can construct a transition matrix related to the dynamical system with T. This matrix has as i,j-entry, i≠j, equal to the sum of the values $T_{ij}$ in the i,j-source of the domain $\Omega_{j}$, while the diagonal entries are equal to the negative of the sum of all the values $T_{ij}$ in all the sinks of the domain $\Omega_{i}$. Nevertheless, there are different situations that lead to the same transition matrix (see Figure 5). For example, consider two domains connected by identical sink–sources, which would lead to a transition matrix equal to the graph Laplacian of two nodes simply connected. This is the same no matter whether the sink and source for each domain are located in the same place or distant from each other, but this leads to different behavior (see [45] for examples on this difference). Thus, not only the dynamical object T is necessary to obtain the behavior of the dynamical process, but also the topology generated by (ω,F).

Second, there exists the possibility that different domains may have different dimensions. Thus, using a metaplex to model classical diffusion leads to the use of the Laplacian in each domain $\Omega_{i}$, but such Laplacians do not behave equally if they are in a 1-dimensional space or in a 3-dimensional one, so the results obtained by using metaplexes are more complex than those obtained by the union of the partial results in each of the considered spaces. For instance, in the field of (continuous) partial differential equations, there are articles showing how mixing domains of different dimensions may lead to different results. An example of this situation can be found in [49], where it is shown that the differential operator arising from the diffusion Laplacian when “stretching” a high-dimensional domain into a 1-dimensional line is not simply the 1-dimensional Laplacian on the line, but an operator that takes into account the topology of the previous high-dimensional domain. Hence, the choices of the operators $H_{i}$ may also model the topological properties of the object that the metaplex is representing. This way, we can use the metaplex to simplify a problem without losing so much information.

## 5. Simplicial Complexes as Metaplexes

**Figure 6:**
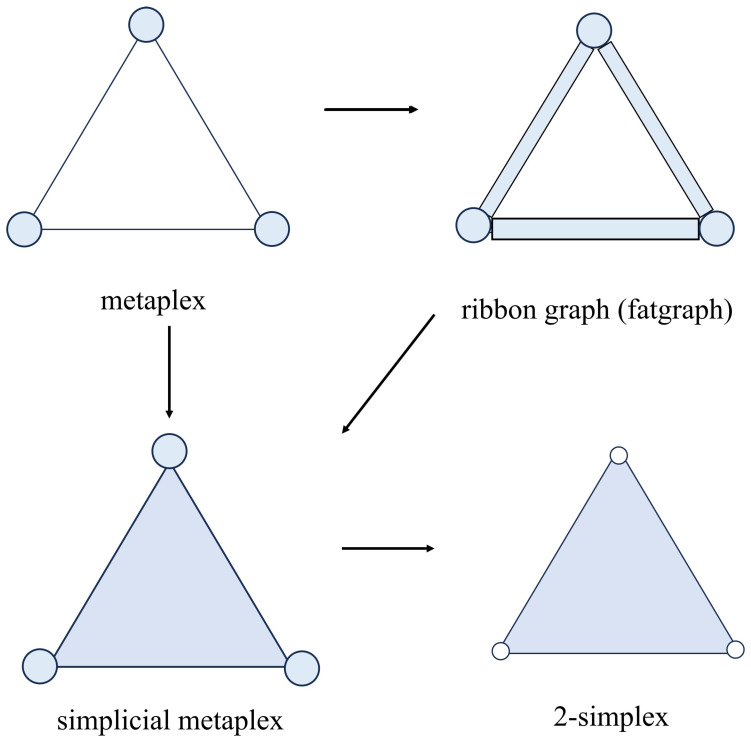
Schematic illustration of the transformations of a metaplex into a simplicial complex. A metaplex can be transformed into a fatgraph by considering its edges as nodes as well, giving them spatial structure. This fatgraph could be transformed into a simplicial metaplex by adding a node for each triangle with the corresponding spatial structure. In the same way, a metaplex can be transformed into a simplicial metaplex by doing both steps at the same time. Finally, a simplicial metaplex can be transformed into a 2-simplex by erasing the spatial structure of all the nodes.

We start here with a metaplex Υ=(V,E,ω,I,F) as defined before. So far, we have closed disks connected by relations. Now, we glue ribbons (topologically another set of closed disks) to the edges of the metaplex, such that the new object is a geometric ribbon graph, also known as fatgraph [50,51]. Notice that ribbon graphs have been previously used to represent certain complex systems such as macromolecules [52]. This new object (see Figure 6) is formally defined as follows.

**Definition** **7.** *A geometric ribbon graph $G=\left(V\left(S\right),E\left(S\right)\right)$ is a surface S with boundary, together with two finite sets of closed disks in S, the set $V\left(S\right)$ of vertices, and a set E(S) of edges. The following restrictions are then imposed:*

1. The surface *S* is covered by the disks of V(S)⋃E(S).
2. The disks intersect only in certain disjoint line segments in *S*;
3. Each such line segment lies in the boundary of one vertex and one edge, and meets no other vertex or edge.
4. Each edge contains exactly two such line segments.

In a similar way, we can glue a triangle along the three edges of the metaplex [53], thus giving rise to a simplicial metaplex (see Figure 6), which can be finally transformed into a simplex by making the vertices structureless points (0-simplices). A fatgraph can also be transformed into a simplicial metaplex by using topological surgery operations for gluing together the three ribbons.

**Figure 7:**
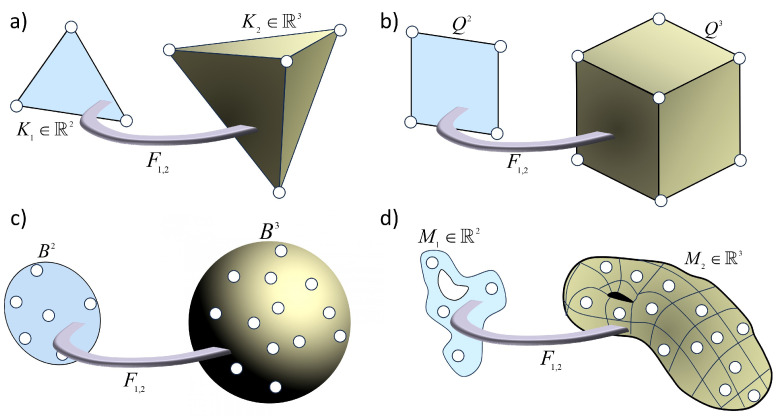
Illustration of different metaplexes formed by 3-dimensional objects which are connected to 2-dimensional ones through their boundary. The dynamical operators are different in each of the objects due to their dimensions. (**a**) A simplicial metaplex formed by two parts $K_{1}$ and $K_{2}$ which consist of a 1-simplex and 2-simplex, respectively. The connection is represented by the map $F_{1,2}$, which, according to Definition 4 acts as a sink in $K_{1}$ and as a source in $K_{2}$. (**b**) A cube complex is a polytopal complex in which $Q^{2}$ and $Q^{3}$ are cubes in 2 and 3 dimensions, respectively. (**c**) An example of CW complex in which sets of nodes are covered by 2- and 3-dimensional balls $B^{2}$ and $B^{3}$ are connected by their boundary spheres. (**d**) An example of a manifold complex formed by two interconnected manifolds (2- and 3-dimensional ones).

To establish a connection between the simplicial complex and the Definition 4 let us consider, for instance, the set $\left(V=\left[0,\ldots ,k\right],E=\{\left[i,j\right]:F_{ij}\neq Ø\}\right)$, and let $\omega ={\left\{\Omega_{i}=K_{i}\right\}}_{i=0}^{k}$ be a set of *n* simplices and I constant. Notice that the dimensions $n_{i}$ are not necessarily the same for every simplex (a condition we have kept in the previous definition of metaplex). Then, simplices are connected via sink and sources, forming a “network” of simplices fulfilling the conditions of Definition 3, and we have a simplicial complex as is illustrated in Figure 7. Additionally, other types of cell complexes are representable as metaplexes. For instance, if we consider groups of nodes that form *k*-cubes instead of simplices, we will be in the presence of a special type of polytopal complex [54] as the one represented in Figure 7. However, the necessity for more flexible representations in which other types of cell complexes are used may emerge from the representation of groups of nodes related by certain important structural characteristics beyond the clique structure. This may include other types of cell complexes used in topology in which groups of nodes related, for instance, by communities are grouped by means of balls or even by manifolds. An example of the first may be the CW complexes [55], which are spaces built up out of balls used as the cells, which are attached step by step through the boundary spheres of the balls. In the second case, an example exists in the particular types of complexes of manifolds, named “complifolds”, proposed by Whitney in 1947 [56], which has received very little attention in the literature. All of these types of complexes are representable by metaplexes. Additionally, metaplexes also allow that each domain $\Omega_{i}\in \omega$ may be of a different type.

### Diffusion on Simplicial Complexes as Metaplexes

**Figure 8:**
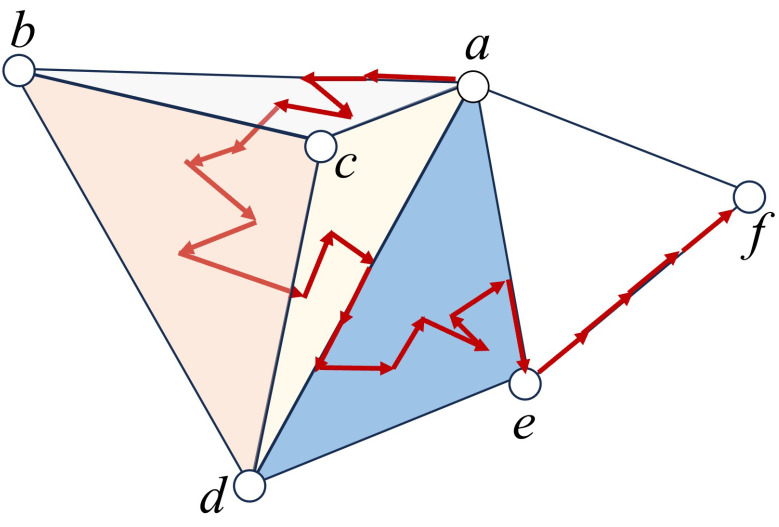
Representation of a simplicial complex consisting of 5 vertices $V=\left\{a,b,c,d,e,f\right\}$ forming a 3-simplex with vertex set $V_{1}=\left\{a,b,c,d\right\},$ a 2-simplex with vertex set $V_{2}=\left\{a,d,e\right\},$ and two 1-simplices with vertex sets $V_{3}=\left\{a,f\right\},$ and $V_{4}=\left\{e,f\right\}$. We illustrate a particle that departs from vertex *a* (a 0-simplex) and performs a 1-dimensional diffusion along the 1-simplex $\left\{a,b\right\}$ which is a face of the triangle $\left\{a,b,c\right\}$ belonging to the 3-simplex. Because only transitions from $R^{m}$ to $R^{m\pm 1}$ inside the same simplex are allowed, the particle hops to the 2-simplex $\left\{a,b,c\right\}$ where it performs a 2-dimensional random walk. From there, it hops to the interior of the 3-simplex and performs a 3-dimensional diffusion, emerging again to one of the faces of the tetrahedra, i.e., the face $\left\{a,c,d\right\}$. The transition from this face can only be either back to the interior of the tetrahedra or to any of the edges of the triangle $\left\{a,c,d\right\}$. Here, the particle hops to the 1-simplex $\left\{a,d\right\}$ from where it can navigate towards the 2-simplex $\left\{a,d,e\right\}$ due to the fact that $\left\{a,d\right\}$ is a simplex shared by both the tetrahedra $\left\{a,b,c,d\right\}$ and the triangle $\left\{a,d,e\right\}.$ From the triangle, it can hop to any of its edges, such as $\left\{a,e\right\}$, hopping through a vertex to the edge $\left\{e,f\right\}$ and ending its long walk at the 0-simplex *f*.

The main advantage of representing simplices and simplicial complexes as metaplexes is that we can preserve a property that underlies the incidence matrices of a simplicial complex: each *n*-dimensional structure can only receive information from n+1- or n−1-dimensional structures. In this way, a change in any of the simplex will affect all of the other elements in the complex, not only those of the same dimension as illustrated in Figure 8. This means that sinks and sources in the simplicial metaplex can only connect adjacent simplices whose dimensions differ by just one. This choice is made here only to keep coherence with previous works in which the incidence matrices were used to form the blocks of the higher-order Laplacian. Although we use this approach to allow a more fair comparison with the previous method, we should notice that the current approach is much more general and allows the definition of transitions between simplices of any dimension, which may correspond to more realistic physical scenarios.

Now, we can choose the dynamical system (Δ,L), consisting of imposing the standard continuous Laplacian [57] in each of the nodes of the metaplex, while the diffusion between nodes is also ruled by the graph Laplacian. Then, for each simplex, we sum the values in the whole domain to obtain a number for each simplex so we can compare the results with those shown in Figure 3. In doing the calculations we will always consider a 0-simplex as a point, just as the node of a graph, a 1-simplex will consist of a 1-dimensional line whose two endpoints are sinks and sources to their respective 0-simplices, and a 2-simplex will be a 2-dimensional equilateral triangle whose edges are all sinks and sources to their respective 1-simplices, which will have a sink and a source consisting on the whole line connected to the triangle, and other two sinks and sources which consist only on the endpoints, which are connected to the 0-simplices that represent the nodes. In this way, the sink and sources are given by the boundaries of the simplices, meaning that the transmission of the information will occur, for example, between a 2-simplex and a 1-simplex, through the shared edge in the 2-simplex. This is also illustrated in Figure 8.

**Figure 9:**
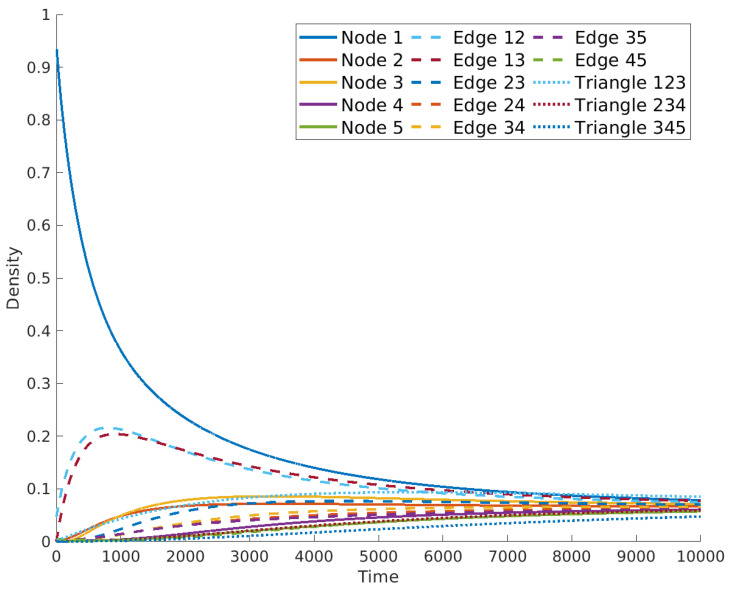
Evolution of the diffusion equation on the simplicial complex in Figure 4 using metaplexes.

In Figure 9, we show the results obtained for the same initial condition used for the results in Figure 3, namely all zeros but on the node 1, the edge (1,2) and the triangle (1,2,3), where the initial concentration is equal to 1 and distributed uniformly for each of the elements. As can be observed, the diffusion on the simplicial complex modeled as a metaplex shows similar behavior to diffusion in both networks or continuous spaces, reaching all domains in the same steady state, which is the average of the initial condition as required in standard diffusion. In this case, it preserves the positivity of the solution, also for the case of high-order simplices. Moreover, it also solves the problem shown in [47], namely the vanishing of the returning time probability when we use the high-order Laplacian to model diffusion on simplicial complexes. In this framework, as the steady state is always positive (provided the initial condition is positive, too), the returning time probability stabilizes for a long time.

## 6. Application

In this section of the paper, we investigate the potential of the simplicial metaplex to study a real-world situation. The macaque visual cortex constitutes a relevant example for several reasons. First, as we have stated before, in brain systems, the presence of volume transmission [35,36,37,38,39] via the extracellular environment makes that different brain regions can be considered as pieces of continuous space filled by neurotransmitters which are diffused by means of blood or cerebrospinal fluid. Second, in the case of the macaque visual cortex, it was recently discovered [58], using functional MRI, the existence of a set of patches that were more active for stimuli containing figures on a background to corresponding control stimuli containing only background, regardless of whether figures were defined by texture, motion, luminance, or disparity. This will allow us to focus on some interesting regions of the simplicial metaplex to try obtaining some valuable information. Let us first describe our numerical setting.

### 6.1. Numerical Setting

Here, we have designed specialized functions accessible within [59]. When it comes to the 2-simplex, we used MATLAB’s PDEModeler TOOLBOX^®^ [60] to generate a mesh discretization of the 2-dimensional equilateral triangle, each of its edges measuring one unit in length. The mesh generated had a total of 210 nodes. Each of the edges of this mesh are composed of 20 nodes. Our representation of the 1-simplex are path graphs also consisting of 21 nodes, mirroring the number of nodes on the edges of the triangle. Finally, the 0-simplex entities have been modeled as point graphs.

A noteworthy adjustment was made to the mass matrix representing the 2-simplex to normalize its area to 1 unit, as opposed to its original value of $\frac{\sqrt{3}}{4}$, which corresponds to the area of an equilateral triangle with unit-length edges. This modification was made to ensure that all elements within the simplicial complexes share an equal measure. It affects the steady state of the diffusive process described before, as it is inversely proportional to the measure of each of the elements in the metaplex. Consequently, not modifying the measure of the 2-simplices would result in the cumulative density within each triangle, which would be different than the cumulative density along the lines or at each node. This choice can be altered depending on the nature of the problem being modeled.

In the process of constructing the sinks and sources within the simplices, we considered a bidirectional flux between entities, meaning that a sink can act as a source and vice versa. Since the discretization of the triangle’s edge has the same number of nodes as the line’s mesh, we connect these nodes one-to-one. In doing so, we are projecting the boundary of the triangle into the line representing the 1-simplices. Similarly, we connect the terminal nodes of the line meshes to the nodes representing the 0-simplices. These choices result in 2-simplices having sink–sources in all their boundaries, while 0-simplices are themselves, as a whole domain, considered as sink–sources. The case of 1-simplices is more complicated since they have two types with sink–source regions. First, their endpoints are connected to the corresponding 0-simplices, and on the other hand, the whole 1-simplex is a sink–source connected to their neighboring 2-simplices, if any.

### 6.2. The Macaque Cortex Network

Here, we simulate how diffusive particles spread inside the macaque visual cortex. Although we do not use any specific parameter for these diffusive particles, we may consider them as a toy model for the diffusion of neurotransmitters in the brain, where both wiring and volume transmission coexist. We use the macaque visual cortex network [61], which consists of 30 nodes representing areas on the macaque’s brain, connected through 190 edges. Using this network as a reference, we constructed the graph of the simplicial network which underlies the metaplex. Such a graph has one vertex for each node in the original network, one vertex for each edge, which is connected to the corresponding nodes, and one vertex for each triangle in the network. When constructing a simplicial complex, it is important to choose which sets of three connected nodes form a 2-simplex or not. In this case, we choose to assign a 2-simplex to every triangle in the network, resulting in 487 triangles in total. We will comment further on these choices at the end of the subsection. We simulated $10^{4}$ time steps from an initial condition consisting of zeros in all the 1 and 2-simplices, and a quantity equal to the degree on the original network in each of the 0-simplices. The initial condition on the nodes is adjusted so that the density transmitted from the 0-simplices to the 1-simplices is the same at the first steps of the simulation.

**Figure 10:**
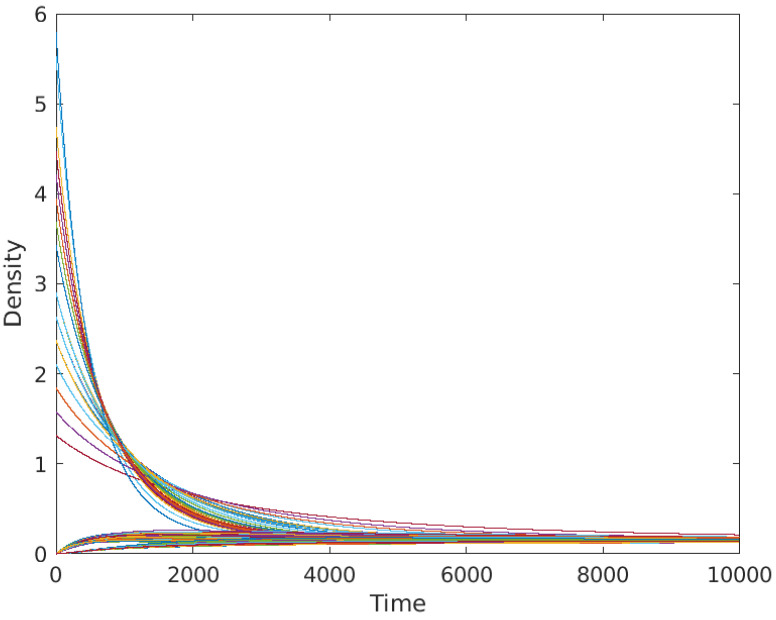
Each line represents the evolution of the concentration of neurotransmitters inside each of the macaque metaplex elements.

**Figure 11:**
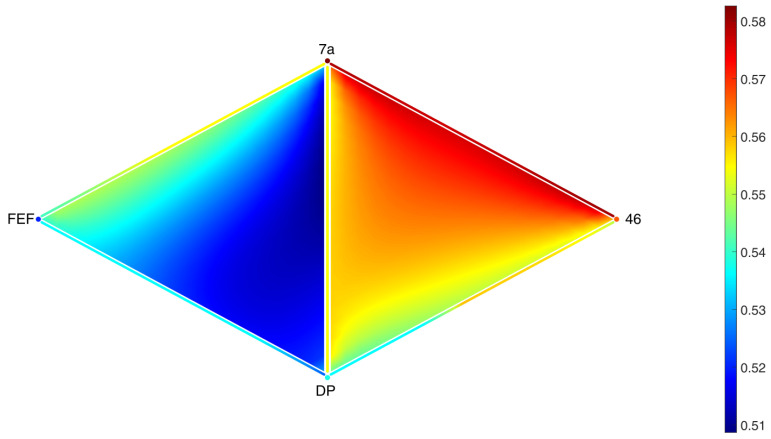
Spatial distribution of the concentration of neurotransmitters along two triangles around the areas 7a, FEF, 46, and DP in the macaque visual cortex after 10,000 time steps.

**Figure 12:**
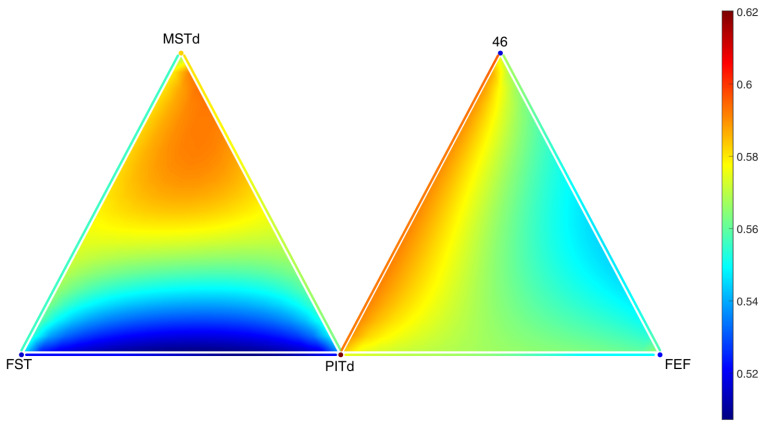
Spatial distribution of the concentration of neurotransmitters along two triangles around the areas MSTd, 46, FST, PITd and FEF in the macaque visual cortex after 10,000 timesteps.

As we can see in Figure 10, the sum of the concentrations over each simplex achieves a uniform steady state. In Figure 11 and Figure 12, it can be seen that the distribution inside different triangles is very different due to the structure of the metaplex. Let us suppose that these triangles were triples of brain regions in which neurotransmitters were diffusing through the extracellular fluid. Then, these results indicate that not all triples of brain regions will display the same concentrations of neurotransmitters. Moreover, these neurotransmitters will not be spread equally across the extracellular fluid covering the triangles. Such features, here analyzed in the form of a toy model, would be very relevant for modeling diffusion in more realistic scenarios.

**Figure 13:**
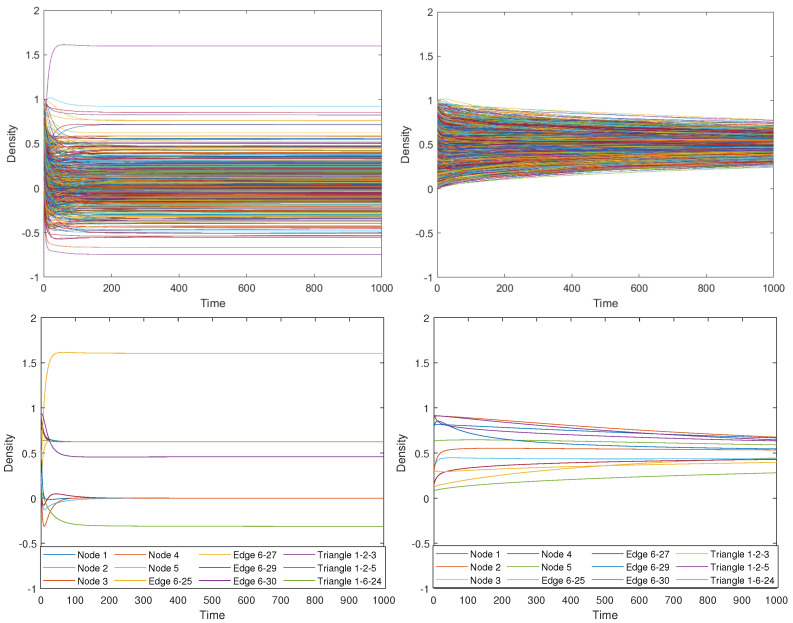
Time evolution of the density of diffusive particles on each of the simplices in the macaque visual cortex. The left figure shows the results of the evolution under the higher-order Laplacian in the simplicial complex, while the right figure plots the evolution using the simplicial metaplexes presented in this article. Lower figures show the density along selected nodes, edges, and triangles. Note that both left figures present non-positive values of the concentration.

Although abstract simplicial complexes are not an appropriate model for this situation, as explained in the Introduction, we can perform simulations using the higher-order Laplacian [47] as the diffusion operator to compare with the results obtained here using the simplicial metaplex. For that purpose, we need to change the initial conditions because, otherwise, the result will be exactly the same as if we perform the simulation on the original graph using the graph Laplacian, not taking advantage of the higher-order structure of the simplicial complex. Hence, we chose random initial conditions all around the simplicial complex to perform this comparison, uniformly distributed for the physical space in the case of the simplicial metaplex. We show the difference in the evolution of the density along the simulation in Figure 13. In this figure, we can observe how the diffusive process using the higher-order Laplacian quickly converges to a non-trivial steady state, which is far from a global consensus. Moreover, most of the simplices have a negative density of particles, which has no physical meaning and reassures that the higher-order Laplacian is not a diffusion operator in the classical way. On the other hand, the current method of diffusion using the simplicial metaplex always shows positive values of the density and converges to the consensus state, as theoretically predicted. Another important difference between the models is that, while the diffusion on the simplicial metaplex is conservative, the process ruled by the higher-order Laplacian is not, and total density in the steady state is not the same as the sum of densities in the initial state. Finally, we observe that the convergence time is much longer in the case of the simplicial metaplex, as the process realistically performs continuous diffusion dynamics inside the simplices and a discrete inter-simplex one.

Another fundamental difference between the way in which higher-order Laplacian and the current approach treat diffusion on simplices is manifested in the following. The diffusion based on higher-order Laplacians treats the whole space inside the simplices as a “patch” in which the concentration of diffusive particles is exactly the same at every part of this space; that is, if we visualize the concentration inside a 2-simplex using this approach we will see a homogeneous color across the whole triangle (the same for a line). As mentioned before, the current approach gives a much more realistic picture in which the concentrations are not distributed homogeneously across the simplices, as we can see in the previous figures.

**Figure 14:**
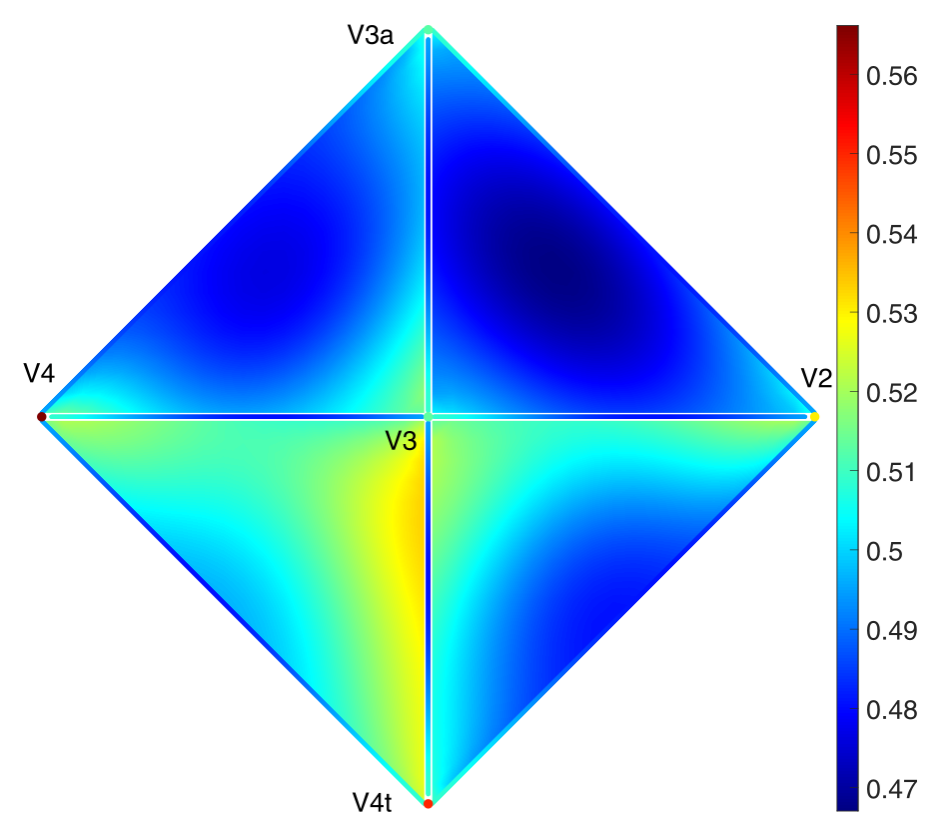
Spatial distribution of the concentration of neurotransmitters in and around the areas V2, V3, V3a, V4, and V4t of the macaque visual cortex after 7500 time steps.

Let us now focus on the areas V2, V3, V3A, V4, and V4A (see Figure 14), which have been recently identified as “more active for stimuli containing figures compared to ground, regardless of whether figures were defined by texture, motion, luminance, or disparity” [58]. As Area V4A is not present in the network from [61], we changed it to Area V4t, which is a nearby area, so that the results from this proof of concept application should be comparable. In Figure 14, we can observe that the potential presence of diffusive particles is not equal around all 5 regions. In particular, diffusive particles are more concentrated in the communication between Regions V3, V4, and V4t. This suggests that volume transmission could play a relevant role in the coactivation of these three areas under certain stimuli.

**Figure 15:**
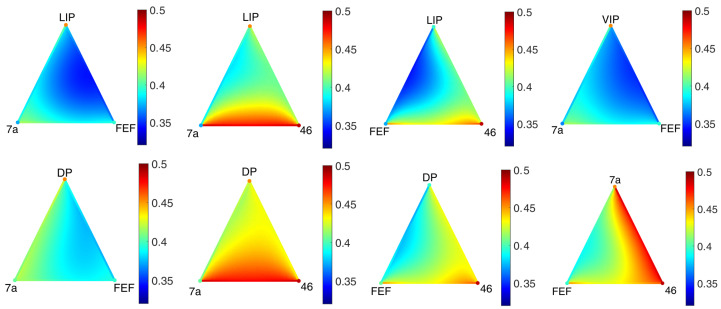
Spatial distribution of the concentration in 8 triangles of the macaque metaplex after 10,000 timesteps.

Although at the center of the 2-simplex V2, V3, and V3A there is significantly less concentration of the diffusive particles than in the regions close to the 1-simplices, the concentration is still relatively high, as there is about 20% less concentration at the center than in the borders. However, there are situations in which the 2-simplices are almost empty. This can be observed, for instance, in Figure 15 in which we illustrate several triangles in the macaque metaplex where the concentration of the diffusive particles is very low almost everywhere in the 2-simplices, but particularly at their central parts.

The first useful insight of this result is that we can use the current method in a self-consistent way; that is, we can start by considering every triangle in the network as a 2-simplex. However, after performing diffusive dynamics in this simplicial metaplex, maybe using a lower resolution to guarantee faster convergence, we can detect such triangles which are almost empty. Then, using a better resolution, we can perform further diffusive dynamics by considering that such triangles are not 2-simplices but holes. Of course, this detection of “holes” is more far reaching than its simple use in improving computational efficiency. For instance, it can be used in the framework of topological data analysis (TDA) [62,63], where there is an interest in finding persistent holes in data [12,64,65,66,67]. Even more, the current approach can be extended to detect holes not only on simplicial complexes but on more general polytopal or CW complexes, which is an interesting line of research nowadays [54].

Secondly, the results obtained through this toy model approach can also be interpreted as indicating the importance of these two methods of transmitting information (VT and WT). Starting with a homogeneously diffusing condition, the structure of the SC appears to favor higher concentrations of neurotransmitters in certain triangles. This suggests that VT may play a crucial role in the connection between such triads of brain regions. Conversely, empty triangles may indicate that considering only WT would be the relevant means of transmitting information between those neurons.

## 7. Conclusions

To advance our understanding of complex systems, we need the appropriate level of sophistication in their representation. Higher-order representation is an important step forward in this direction. In particular, simplicial complexes allow us to capture unique features that combine the geometric and topological features of some complex systems. However, this representation is not appropriate for any kind of complex system, particularly when what we are interested in is the dynamics taking place on it. In those cases where the simplicial complex representation is needed, the proper existence of local continuous spaces interconnected by discrete relations challenges our models to describe dynamics on them.

We have considered here an extension of the concept of metaplexes previously defined to account for geometric simplicial complexes and (diffusive) dynamics on them. Using these simplicial metaplexes we have solved the problem of diffusion on these representations of complex systems and resolved some of the problems found with previous models. The diffusion model on simplicial metaplexes allows us to uniquely trace the concentration of diffusive particles across the continuous spaces of the simplices in any dimension while maintaining the discrete navigation between the simplices.

The formal definition of simplicial metaplexes and of dynamical systems on them allow several further avenues for the study of complex systems. For instance, although not limited to them we can mention: (i) extension to other dynamics, e.g., synchronization, reaction–diffusion; (ii) extension to other types of continuous spaces beyond simplices, e.g., polytopal and CW complexes, manifolds, etc.; (iii) changing the sinks and sources from being the common faces between simplices to be particular regions inside them, allowing transitions between simplices of different dimensions.

## Data Availability

The codes used to generate the results in this article can be found in [59]. More codes generated or used during this study are available from the corresponding author upon request.

## References

[1] Siegenfeld A.F., Bar-Yam Y. (2020). An introduction to complex systems science and its applications. Complexity.

[2] Thurner S., Hanel R., Klimek P. (2018). Introduction to the Theory of Complex Systems.

[3] Estrada E. (2023). What is a complex system, after all?. Found. Sci..

[4] Torres L., Blevins A.S., Bassett D., Eliassi-Rad T. (2021). The why, how, and when of representations for complex systems. SIAM Rev..

[5] Boccaletti S., Latora V., Moreno Y., Chavez M., Hwang D.U. (2006). Complex networks: Structure and dynamics. Phys. Rep..

[6] Estrada E. (2012). The Structure of Complex Networks: Theory and Applications.

[7] Holme P., Saramäki J. (2012). Temporal networks. Phys. Rep..

[8] Boccaletti S., Bianconi G., Criado R., Del Genio C.I., Gómez-Gardenes J., Romance M., Sendina-Nadal I., Wang Z., Zanin M. (2014). The structure and dynamics of multilayer networks. Phys. Rep..

[9] Kivelä M., Arenas A., Barthelemy M., Gleeson J.P., Moreno Y., Porter M.A. (2014). Multilayer networks. J. Complex Netw..

[10] Wasserman S., Faust K. (1994). Social Network Analysis: Methods and Applications.

[11] Estrada E., Rodríguez-Velázquez J.A. (2006). Subgraph centrality and clustering in complex hyper-networks. Phys. A Stat. Mech. Its Appl..

[12] Salnikov V., Cassese D., Lambiotte R. (2018). Simplicial complexes and complex systems. Eur. J. Phys..

[13] Giusti C., Ghrist R., Bassett D.S. (2016). Two’s company, three (or more) is a simplex: Algebraic-topological tools for understanding higher-order structure in neural data. J. Comput. Neurosci..

[14] Estrada E., Ross G.J. (2018). Centralities in simplicial complexes. Applications to protein interaction networks. J. Theor. Biol..

[15] Bianconi G. (2015). Interdisciplinary and physics challenges of network theory. Europhys. Lett..

[16] Bick C., Gross E., Harrington H.A., Schaub M.T. (2023). What are higher-order networks?. SIAM Rev..

[17] Majhi S., Perc M., Ghosh D. (2022). Dynamics on higher-order networks: A review. J. R. Soc. Interface.

[18] Bianconi G. (2021). Higher-Order Networks.

[19] Benson A.R., Gleich D.F., Leskovec J. (2016). Higher-order organization of complex networks. Science.

[20] Lambiotte R., Rosvall M., Scholtes I. (2019). From networks to optimal higher-order models of complex systems. Nat. Phys..

[21] Battiston F., Cencetti G., Iacopini I., Latora V., Lucas M., Patania A., Young J.G., Petri G. (2020). Networks beyond pairwise interactions: Structure and dynamics. Phys. Rep..

[22] Zhang Y., Lucas M., Battiston F. (2023). Higher-order interactions shape collective dynamics differently in hypergraphs and simplicial complexes. Nat. Commun..

[23] Newman M.E. (2004). Coauthorship networks and patterns of scientific collaboration. Proc. Natl. Acad. Sci. USA.

[24] Kumar S. (2015). Co-authorship networks: A review of the literature. Aslib J. Inf. Manag..

[25] Wang D., Zhao Y., Leng H., Small M. (2020). A social communication model based on simplicial complexes. Phys. Lett. A.

[26] Atkin R.H. (1972). From cohomology in physics to q-connectivity in social science. Int. J. Man-Mach. Stud..

[27] Atkin R.H. (1974). Mathematical Structure in Human Affairs.

[28] Seidman S.B. (1981). Structures induced by collections of subsets: A hypergraph approach. Math. Soc. Sci..

[29] Freeman L.C., White D.R. (1993). Using Galois lattices to represent network data. Sociol. Methodol..

[30] Young J.G., Petri G., Peixoto T.P. (2021). Hypergraph reconstruction from network data. Commun. Phys..

[31] Myers A., Joslyn C., Kay B., Purvine E., Roek G., Shapiro M. (2023). Topological analysis of temporal hypergraphs. Proceedings of the International Workshop on Algorithms and Models for the Web-Graph.

[32] Iacopini I., Petri G., Barrat A., Latora V. (2019). Simplicial models of social contagion. Nat. Commun..

[33] Maletić S., Rajković M. (2014). Consensus formation on a simplicial complex of opinions. Phys. A Stat. Mech. Its Appl..

[34] Nie Y., Li W., Pan L., Lin T., Wang W. (2022). Markovian approach to tackle competing pathogens in simplicial complex. Appl. Math. Comput..

[35] Agnati L.F., Guidolin D., Guescini M., Genedani S., Fuxe K. (2010). Understanding wiring and volume transmission. Brain Res. Rev..

[36] Fuxe K., Borroto-Escuela D.O., Tarakanov A., Fernandez W.R., Manger P., Rivera A., van Craenenbroeck K., Skieterska K., Diaz-Cabiale Z., Filip M. (2013). Understanding the balance and integration of volume and synaptic transmission. Relevance for psychiatry. Neurol. Psychiatry Brain Res..

[37] Taber K.H., Hurley R.A. (2014). Volume transmission in the brain: Beyond the synapse. J. Neuropsychiatry Clin. Neurosci..

[38] Sykova E. (2004). Extrasynaptic volume transmission and diffusion parameters of the extracellular space. Neuroscience.

[39] Szapiro G., Barbour B. (2007). Multiple climbing fibers signal to molecular layer interneurons exclusively via glutamate spillover. Nat. Neurosci..

[40] Berge C. (1984). Hypergraphs: Combinatorics of Finite Sets.

[41] Naber G.L. (1980). Topological Methods in Euclidean Spaces.

[42] Hatcher A. (2002). Algebraic Topology.

[43] Lee J. (2010). Introduction to Topological Manifolds.

[44] Lotito Q.F., Musciotto F., Montresor A., Battiston F. (2022). Higher-order motif analysis in hypergraphs. Commun. Phys..

[45] Estrada E., Estrada-Rodriguez G., Gimperlein H. (2020). Metaplex networks: Influence of the exo-endo structure of complex systems on diffusion. SIAM Rev..

[46] Masuda N., Porter M.A., Lambiotte R. (2017). Random walks and diffusion on networks. Phys. Rep..

[47] Torres J.J., Bianconi G. (2020). Simplicial complexes: Higher-order spectral dimension and dynamics. J. Phys. Complex..

[48] DiBenedetto E. (2009). Partial Differential Equations.

[49] Arrieta J.M., Carvalho A.N., Lozada-Cruz G. (2006). Dynamics in dumbbell domains I. Continuity of the set of equilibria. J. Differ. Equ..

[50] Bollobás B., Riordan O. (2002). A polynomial of graphs on surfaces. Math. Ann..

[51] Ellis-Monaghan J.A., Moffatt I. (2013). Graphs on Surfaces: Dualities, Polynomials, and Knots.

[52] Carson M. (1987). Ribbon models of macromolecules. J. Mol. Graph..

[53] Bohman T., Frieze A., Lubetzky E. (2015). Random triangle removal. Adv. Math..

[54] Singh R.P., Wilsey P.A. Polytopal Complex Construction and Use in Persistent Homology. Proceedings of the 2022 IEEE International Conference on Data Mining Workshops (ICDMW).

[55] Lundell A.T., Weingram S. (2012). The Topology of CW Complexes.

[56] Whitney H. (1947). Complexes of manifolds. Proc. Natl. Acad. Sci. USA.

[57] Evans L.C. (2022). Partial Differential Equations.

[58] Hesse J.K., Tsao D.Y. (2023). Functional modules for visual scene segmentation in macaque visual cortex. Proc. Natl. Acad. Sci. USA.

[59] Miranda Barrado M., Estrada-Rodríguez G., Estrada E. (2023). Simplicial-Metaplexes. GitHub Repo. https://github.com/ManuM2B/Simplicial-Metaplexes.git.

[60] The MathWorks Inc. (2022). Partial Differential Equation Toolbox, Version: 23.2 (R2022b).

[61] Sporns O., Kötter R. (2004). Motifs in brain networks. PLoS Biol..

[62] Wasserman L. (2018). Topological data analysis. Annu. Rev. Stat. Its Appl..

[63] Zomorodian A. (2012). Topological data analysis. Adv. Appl. Comput. Topol..

[64] Gowdridge T., Dervilis N., Worden K. (2022). On Topological Data Analysis for SHM: An Introduction to Persistent Homology. Proceedings of the Data Science in Engineering, Volume 9: Proceedings of the 39th IMAC, a Conference and Exposition on Structural Dynamics.

[65] Carlsson E., Carlsson J.G., Sweitzer S. (2022). Applying topological data analysis to local search problems. Found. Data Sci..

[66] Phinyomark A., Ibáñez-Marcelo E., Petri G. (2018). Topological Data analysis of Biomedical Big Data. Signal Processing and Machine Learning for Biomedical Big Data.

[67] Billings J., Saggar M., Hlinka J., Keilholz S., Petri G. (2021). Simplicial and topological descriptions of human brain dynamics. Netw. Neurosci..

